# Shaping the Future of AI in Organ Transplantation: Position Paper of the European Society for Organ Transplantation

**DOI:** 10.3389/ti.2026.16316

**Published:** 2026-04-20

**Authors:** Georgios Kourounis, Stephen Gilbert, Simon R. Knight, Amanda Leal, Jackie Leach Scully, Alexandre Loupy, Dominique E. Martin, Evgenia Preka, Nadia Primc, Fernando Seoane Martinez, Helena Webb, Gabriel C. Oniscu, Colin Wilson

**Affiliations:** 1 NIHR Blood and Transplant Research Unit in Organ Donation and Transplantation, Newcastle University and Cambridge University, Newcastle upon Tyne, United Kingdom; 2 Institute of Transplantation, The Freeman Hospital, Newcastle upon Tyne, United Kingdom; 3 Else Kröner Fresenius Centre for Digital Health, TUD Dresden University of Technology, Dresden, Germany; 4 Nuffield Department of Surgical Sciences, University of Oxford, Oxford, United Kingdom; 5 The Global Agency for Responsible AI in Health, Geneva, Switzerland; 6 Disability Innovation Institute, University of New South Wales, Sydney, NSW, Australia; 7 Université Paris Cité, INSERM U970, Paris Institute for Transplantation and Organ Regeneration, Paris, France; 8 School of Medicine, Deakin University, Geelong, VIC, Australia; 9 Institute of History and Ethics of Medicine, Medical Faculty, Heidelberg University, Heidelberg, Germany; 10 Department of Clinical Science, Intervention and Technology, Karolinska Institutet, Stockholm, Sweden; 11 Department of Clinical Physiology, Karolinska University Hospital, Stockholm, Sweden; 12 Department of Textile Technology, Faculty of Textiles, Engineering and Business Swedish School of Textiles, University of Borås, Borås, Sweden; 13 Department of Medical Technologies, Karolinska University Hospital, Huddinge, Sweden; 14 School of Computer Science, University of Nottingham, Nottingham, United Kingdom; 15 Division of Transplantation Surgery, CLINTEC, Karolinska Institutet, Stockholm, Sweden

**Keywords:** artificial intelligence (AI), ESOT, machine learning, organ transplantation (OT), position paper

## Abstract

Advances in AI hold considerable promise for organ transplantation. While every transformation brings change, not all change is transformative. Despite the rapid growth of AI in medicine, most applications remain in developmental or experimental stages, with relatively few having been successfully integrated into routine clinical practice. As a professional society, ESOT recognises that achieving meaningful impact will require more than technical progress. This position paper outlines five critical domains for successful implementation. (1) High-quality development: Coordinated collaboration and methodological rigour are prerequisites for trust; AI is only as robust as the data used to train it. (2) Ethical considerations: We must address risks to equity and access to care, and move from generic ethical principles to transplantation-specific ethical guidance. (3) Regulatory landscape: AI in transplantation is regulated under both EU medical device and AI legislation; compliance is central to stakeholder trust. (4) Responsible adoption: AI should augment, not replace, human expertise. Strengthening AI literacy is essential for meaningful adoption. (5) Participatory design: Active involvement of transplant professionals and patients is essential to address real clinical needs. These statements serve as a strategic framework to guide clinicians, researchers, and policymakers in making AI a genuine force multiplier for the transplant community.

## Introduction

Artificial intelligence (AI) is increasingly recognised as a highly disruptive technology with the capacity to address challenges across multiple domains of medicine. Broadly defined, AI refers to computational systems that perform tasks traditionally requiring human intelligence. These include learning from data, recognising patterns, making predictions, and supporting complex decisions. In healthcare, AI encompasses a diverse set of tools, including supervised and unsupervised machine learning, natural language processing, computer vision, and generative models. These technologies process large datasets, support clinical judgement, and enhance precision in diagnosis, treatment, and follow-up.

The development of AI in medicine has gained considerable momentum in recent years. Improvements in model performance, the availability of large-scale data, and increased computational power have accelerated both academic and industry-driven research [1, 2]. In transplantation, emerging AI applications span donor–recipient matching [3–5], outcome prediction [6–10], computer vision tools [11–16], and generative AI [17]. Despite rapid progress, relatively few of these applications have so far been integrated into routine clinical practice.

As the European Society for Organ Transplantation (ESOT), we recognise both the considerable promise and the set of challenges that accompany the advancement of AI in our field. AI could contribute along the entire transplant pathway by supporting donor–recipient matching and organ allocation, guiding organ acceptance decisions, and enhancing risk stratification and follow-up after transplantation, effectively moving from an office-based model of care delivery to continuous monitoring, placing the patient at the centre of post-transplant care [18]. While AI offers clear opportunities to improve patient outcomes, streamline clinical workflows, and advance healthcare equity, the path to meaningful adoption must be balanced against complex technical, regulatory, and ethical considerations. Similar themes are emerging across other medical disciplines, although transplantation presents distinct challenges related to organ scarcity, allocation, and longitudinal outcome assessment [19, 20]. Ensuring that AI adoption serves the interests of patients and clinicians is paramount.

ESOT is committed to taking an active role in shaping the future of AI in transplantation. Achieving positive and impactful progress in transplantation will require strategic alignment among all stakeholders, including patients, clinicians, researchers, policymakers, regulators, and industry partners. Building a future where AI can be a true force multiplier in transplantation will depend on collaboration, transparency, and a shared commitment to ethical, patient-centred innovation.

The position statements aim to identify how current opportunities can be realised most effectively while navigating the technical, ethical, and regulatory challenges that may hinder meaningful implementation. By outlining these considerations, the paper seeks to provide a clear and pragmatic framework to support responsible, patient-centred AI adoption across the transplant pathway.

## Development of the ESOT AI Position Statements

The ESOT AI Working Group was convened to produce position statements to guide the adoption of AI in transplantation. The multidisciplinary team comprised physicians and surgeons involved in transplantation, AI and data science experts, bioethicists, transplant recipients and patient representatives, as well as policy and regulatory experts in digital health. The recommendations and position statements were developed iteratively through a series of structured virtual and in-person discussions, including a focused in-person meeting at the ESOT 2025 Congress in London. The process was designed to identify key domains and produce a framework for responsible AI adoption in transplantation. This was not a formal Delphi process, and no predefined voting threshold was used. Instead, statements were refined through repeated group discussion until agreement was reached on their wording and scope.

In parallel with the expert consensus process, ESOT conducted a survey of its membership to explore familiarity with AI, current use of AI tools, and perceived opportunities and concerns regarding AI in transplantation. The survey findings were used to contextualise the consensus statements and to assess their alignment with the views of the wider ESOT membership. Overall, survey responses were consistent with expert opinion. The survey questionnaire and summary of the results are provided in the Supplementary Material SA1, 2.

**FIGURE 1:**
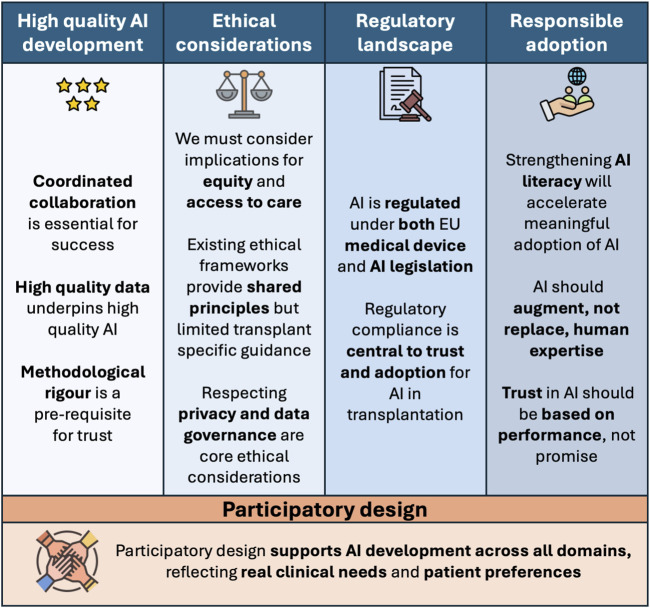
Overview of ESOT position statements on artificial intelligence in transplantation. Four domains—high-quality AI development, ethical considerations, the regulatory landscape, and responsible adoption—summarise key considerations for implementation of AI in transplantation. The fifth domain, participatory design, underpins all domains, ensuring that AI development reflects real clinical needs and patient preferences.

To structure the recommendations and position statements, five domains were chosen reflecting the key requirements and challenges relevant to AI in transplantation: high-quality AI development and validation, ethical considerations, the regulatory landscape, responsible adoption, and participatory design (Figure 1).

## High-Quality AI Development and Validation

Recommendation 1: Clinicians collecting or providing data for use by AI algorithms should ensure that the data are accurate, valid, correctly labelled, comprehensive, unaffected by selection bias, and stored securely.

### Coordinated Collaboration Is Essential for Developing Robust AI

AI innovation will not succeed in isolation. High-quality AI development in transplantation depends on coordinated collaboration across clinical, scientific, and institutional boundaries. Given the complexity and high-stakes nature of donation and transplantation, AI systems developed in isolation are unlikely to generate sufficiently robust or generalisable evidence to support clinical use [21]. Although single-centre studies may offer early insights, multi-centre collaborations are necessary to enable external validation and, where feasible, provide prospective evaluation across populations and practice settings [22, 23].

Collaboration is also critical for curating datasets of sufficient scale and representativeness. Although several data-sharing initiatives and open-science portals now support AI research, few currently include transplantation-specific data [24, 25]. ESOT has already taken steps to address this by creating a pan-European registry platform, supported by the European Commission, that will host data on both transplant recipients and living donors [26]. Finally, parallel research in synthetic data generation offers another potential route to mitigating data scarcity without compromising patient privacy [27, 28].

### High-Quality AI Relies on High-Quality Data

AI tools are only as robust as the datasets used to train them. Although advanced algorithms can identify complex patterns within large datasets, they cannot compensate for poor data quality, inconsistency, or systematic bias [29]. In transplantation, these challenges are pronounced due to variations in clinical practice, donor and recipient characteristics, and allocation processes across centres, regions, and health systems [21]. Without careful curation and transparent reporting, such heterogeneity risks producing AI models that perform well in development settings but fail to generalise in practice [30].

Data governance requirements introduce additional and unavoidable complexity. Strong safeguards are essential to protect patient privacy and maintain public trust. Yet restrictive data-sharing frameworks can unintentionally reinforce fragmented, single-centre datasets and limit opportunities for validation and collaboration. The twin challenges of heterogeneity and restricted access must be addressed in parallel. Well-assembled data that is inaccessible cannot drive progress, just as open, poor-quality data cannot yield trustworthy insights.

### Reliable Evidence Is a Prerequisite for AI to Be Trusted in Transplantation

Methodological rigour is essential at all stages of healthcare AI development, as these tools will support decisions that are high-stakes, time-sensitive, and often irreversible. Evidence must therefore demonstrate not only technical performance but also reliability, generalisability, and clinical relevance across diverse populations and practice settings.

Addressing the diversity of research requirements will require the use of multiple study designs. Registry-based analyses offer scale, longitudinal follow-up, and real-world relevance. Still, they are frequently affected by missing data, heterogeneity, and unmeasured confounding, which can limit model validity if not appropriately addressed [31]. Prospective studies and trials provide more controlled evaluation but are resource-intensive and may not fully reflect real-world complexity. Across study designs, transplantation AI studies should report not only discrimination, but also calibration, external validation, subgroup performance, and the handling of missing data [32, 33]. Where relevant, analyses should also account for variation between centres which may substantially influence model performance and transportability.

Transparent reporting of data provenance, handling of “missingness,” and clarity about sources of bias are therefore critical, regardless of study design. To support consistent evaluation and informed clinical decision-making, AI research in transplantation should adhere to established, evidence-based frameworks for development and reporting, including TRIPOD + AI, DECIDE-AI, and CONSORT-AI [32, 34, 35]. Alignment with such standards is necessary to enable critical appraisal and to distinguish exploratory research from tools sufficiently mature for clinical use. Without this level of methodological transparency and rigour, AI systems risk undermining trust, hindering responsible adoption in practice.

## Ethical Considerations

Recommendation 2: To use AI tools responsibly, transplant clinicians should be familiar with the international guidelines on the ethical use of AI and understand how their patients' data will be protected.

### AI in Transplantation Introduces Ethical Considerations for Equity and Access to Care

The design, development, and deployment of AI in transplantation raise profound ethical considerations. Central among these is the risk of bias. Where an inadequately diverse dataset is used to train an AI algorithm, the system’s outputs may be negatively affected, leading to inaccurate outcomes and/or systematic discrimination when applied in particular populations. Examples include predictive modelling tools that provide inaccurate information for specific socio-economic and racial groups [36, 37]. Bias may arise at multiple stages of the AI lifecycle, including data collection, labelling, feature selection, and deployment, and its effects often disproportionately impact populations that are already marginalised or underserved [37, 38].

In transplantation, these risks are significant given the scarcity of deceased donor organs, the high stakes of allocation decisions, and the potential for AI outputs to influence access to life-saving treatments. Ethical concerns also arise in decisions about where and how AI systems are deployed, which populations benefit from their use, and how their impacts are monitored over time. Together, these issues underscore the need for explicit ethical scrutiny of AI systems used in transplantation.

### Existing Ethical AI Frameworks Provide Shared Principles but Limited Transplantation-Specific Guidance

The landscape of ethical AI guidelines is characterised by a convergence of international, regional, and organisational frameworks that share core principles while emphasising distinct implementation approaches. Despite the large number of guidelines, there is striking overlap in the principles and themes they address, namely 1) transparency and explainability, 2) accountability, 3) fairness and non-discrimination, 4) privacy and data protection, 5) safety and reliability, and 6) human oversight, which is often considered a prerequisite for the ethically justified use of AI in healthcare. An overview of published ethical AI guidelines relevant to healthcare and transplantation is summarised in Table 1.

**TABLE 1:** Overview of international, regional, and organisational ethical frameworks relevant to the development and use of artificial intelligence in healthcare.

Issuing body	Guideline
United Nations Educational, Scientific and Cultural Organisation (UNESCO)	AI Ethics Recommendation [39]
World Health Organisation (WHO)	Guidance on Ethics and Governance of Artificial Intelligence for Health [40]
European Union (EU)	Ethics Guidelines for Trustworthy AI [41]
European Union (EU)	EU AI Act Regulation (EU) [30]
Organisation for Economic Co-operation and Development (OECD)	AI Principles [42]
Institute of Electrical and Electronics Engineers (IEEE)	IEEE Ethically Aligned Design [43]
Microsoft	Responsible AI Standard [44]
Google	AI Principles [45]
National ethical framework – Australia	AI Ethics Principles [46]
National ethical framework – USA	National Institute of Standards and Technology (NIST) AI Risk Management Framework [47]

Most guidance remains high-level, lacking detail and specificity, perhaps reflecting the nascent stage of AI development as various technologies evolve from the innovation stage and become more widely implemented. High-level guidance stating the need to ensure distributive justice by avoiding bias, for example, does not help identify the practical steps to be taken by any sector, profession, or particular technology. The objectives of AI ethics must shift from provision of generic guidance to development of context-specific and implementable guidance to address real problems/ethical concerns impacting clinical practice and patient outcomes.

This is particularly important in the context of organ donation and transplantation systems, since their unique ethical complexity may be exacerbated by the introduction of AI. Here, an essential first step will be to identify and characterise the challenges. A non-exhaustive list of examples includes: bias in algorithmic organ allocation; the potential effect of AI use on public willingness to donate; and how to mitigate the known underrepresentation of specific populations, such as ethnic minorities, in transplantation databases [48–50].

### Respecting Privacy and Data Rights Are Core Ethical Considerations

Since AI development relies on the collection and processing of large datasets, data security and privacy are core ethical concerns. When large amounts of personal data are collected and processed their secure storage, appropriate anonymisation for confidentiality, and alignment with data protection standards present difficulties [51]. The growing volume of data collection and secondary data use can challenge individuals’ ability to understand, control, and consent to how their information is shared [52]. In transplantation, where public trust underpins both donation and long-term engagement with care, perceived failures in data stewardship risk undermining confidence in AI-enabled practices.

Ethical data governance therefore extends beyond legal compliance to include transparency about data use, proportionality in data collection, and accountability for downstream impacts. The challenge is to balance the societal value of data-driven innovation with respect for individual rights and expectations, particularly in a field where data originates with vulnerable patients and incapacitated donors. Failure to address these ethical dimensions risks eroding trust and limiting the potential of AI applications in transplantation.

## Regulatory Landscape

Recommendation 3: Clinicians should ensure that AI tools used in routine clinical practice have undergone appropriate regulatory approval.

### AI in Transplantation Is Regulated Under Both EU Medical Device and AI Legislation

In the European Union (EU), most AI applications relevant to organ donation and transplantation will be regulated as medical device software and, with rare exceptions, as high-risk AI systems. Although the EU has long had a regulatory framework for the medical applications of AI, it is rapidly evolving. This is in part due to the planned introduction of new AI legislation and standards, and in part to the landscape which develops behind technologies submitted for approval.

The EU’s AI regulatory framework is now both multi-layered and intersecting. It combines the “vertical”, product-specific Medical Device Regulation (MDR), (Regulation (EU) 2017/745) [53] with the recent “horizontal” EU AI Regulation (Regulation (EU) 2024/1689) [54]. The latter is the world’s first comprehensive AI law, establishing a risk-based classification system that spans all product domains, including AI-enabled tools classified as medical device software (MDSW). AI systems used in healthcare are classified as ‘high-risk’ if they require third-party assessment under the MDR (or the related *In Vitro* Diagnosis Devices Regulation (IVDR), Regulation (EU) 2017/746) [55].

In practice, most AI-enabled medical devices require such third-party assessment, including all medical software used for individual patient diagnosis and therapy, and all software used for tissue typing of blood, tissues, or organs intended for transfusion or transplantation. The MDR applies to all new medical devices entering the market and to all MDSW from no later than 31 December 2028, while the AI Act applies from no later than 2 August 2027.

An unresolved challenge is regulating adaptive AI systems that learn from new data after deployment. Current regulatory frameworks are built around “locked” models whose performance is fixed at the point of release. These models are controlled via version releases and formal change management. Consequently, AI-enabled medical devices are authorised as static algorithms with periodic, regulator-reviewed updates rather than continuously learning systems operating in real time. Moving forward, we require clearer approaches to post-deployment monitoring and real-world performance evaluation, including the use of registry-based data where appropriate. Such mechanisms may help detect performance drift, assess real-world safety and effectiveness, and provide ongoing assurance after deployment. However, regulatory expectations for these lifecycle issues, particularly for adaptive models, remain incompletely defined and continue to evolve. Given the rapid pace of AI development, we need pathways that accommodate adaptive AI tools so that governance keeps pace with real-world innovation.

Taken together, the EU AI Regulation and the MDR require compliance with strict requirements for both the developers and deployers (i.e., the health system that makes the AI system available to end users). These obligations cover core principles such as the *General Safety and Performance Requirements* (MDR), which mandate a highly prescriptive medical device quality management system and detailed technical documentation. Furthermore, the AI Regulation introduces requirements for *Data Governance* (ensuring high-quality, representative datasets to avoid bias) and mandates *Transparency and Human Oversight* (designing systems to be explainable and subject to human control). To demonstrate compliance with both regulations, most AI-enabled transplant-related devices must undergo a joint formal conformity assessment by a notified body before they can be placed on the market or put into service. This dual approach ensures that AI applications in medicine are transparent, ethical, accountable, safe, and effective.

### Regulatory Compliance Is Central to Trust and Adoption of AI Systems in Transplantation

The need for robust regulatory compliance cannot be overstated. For any AI technologies to be successfully adopted across any medical field, they must first gain the trust of all stakeholders: decision-makers, physicians, surgeons, nurses, patients, and the public. For transplant teams, the knowledge that an AI system has successfully navigated the regulatory pathway provides assurance that it was developed using appropriate quality and safety management processes. These processes are designed to ensure that data used for training and validation are of sufficient quality and that foreseeable risks, including those related to bias and error, are identified and mitigated.

Safe implementation and knowledge exchange across centres will also require greater consistency in terminology, evaluation criteria, and monitoring strategies. Depending on the intended use-case, this may include clearly defined inputs and outputs, structured thresholds for action, prospective monitoring of performance after deployment, and processes for review, recalibration, or suspension if performance drift or safety concerns emerge [56].

Compliance with the AI Act also requires that high-risk AI systems incorporate meaningful human oversight and are both tested and documented in ways that support traceability and audit. This is especially important for AI tools whose internal workings may be difficult to interpret. While regulation does not eliminate all concerns about opacity, it does require manufacturers to provide sufficient information about system behaviour, limitations, and conditions of use to enable informed clinical deployment and to support investigation of adverse events. In effect, the regulatory framework provides a structured basis for accountability when AI systems are integrated into complex decision-making processes such as transplantation.

## Responsible Adoption

Recommendation 4: Transplantation professionals need a role- appropriate understanding of what an AI system is designed to do, the data sources and cohort groups used to develop it, and its strengths and limitations.

### Strengthening AI Literacy Will Accelerate Meaningful Adoption of AI

Improved digital and AI literacy is a fundamental prerequisite for the responsible implementation of AI in transplantation. Transplant clinicians and trainees must be equipped to appraise AI-based tools critically and to recognise when use of “AI” adds clinical value. However not everyone needs to become a technical AI expert. The accepted standard should be that transplantation professionals understand how a tool was developed and its expected strengths and limitations when applied to their patients [57].

This level of literacy will enable clinicians to better interpret and implement AI-generated outputs within their specific clinical contexts. It will also enable clinicians to act as informed intermediaries between AI systems and patients, explaining in clear terms the role of AI in their care without overstating its capabilities. In transplantation, where trust underpins both donation and recipient care, AI literacy should become a practical requirement for transplantation professionals rather than a special interest for enthusiasts.

### AI in Transplantation Should Augment, Not Replace, Human Expertise

The increasing availability of decision-support tools raises important concerns about how clinicians interact with AI systems. Automation bias, in which clinicians defer to algorithmic outputs over other available evidence, can occur when an AI-generated recommendation is perceived as more objective or defensible than personal clinical judgment, even when the latter may be superior [44]. This risk is heightened for less experienced clinicians and in time-pressured or complex situations, where there may be a stronger temptation to follow the system rather than question it [58].

In parallel, the growing reliance on algorithmic assistance raises new concerns about deskilling—the potential erosion of clinical ability as tasks get increasingly delegated to AI [59]. Patterns of “deskilling,” “mis-skilling,” or even “never-skilling” may emerge if clinicians rely on AI before core competencies are fully developed. Safeguarding against this will require deliberate strategies, such as structured training that maintains hands-on clinical judgment, critical appraisal of AI outputs, and periodic “AI-off” practice to preserve independent reasoning [60]. Ensuring that AI augments rather than replaces human expertise will be essential for sustaining confidence, competence, and quality of care.

### Trust in AI Systems Should Be Based on Performance, Not Promise

AI is increasingly touted in healthcare, yet the term “AI” is often overused and poorly defined, with many systems branded as “AI” offering little advantage over traditional analytics [61–63]. Tools should demonstrate strong performance for their intended task, with results reported across clinically relevant subgroups, including those often underrepresented in training data. Developers should actively look for performance gaps, communicate where predictions are less reliable, and, where possible, mitigate these limitations. These expectations are consistent with the ethical and regulatory principles outlined above, including attention to bias, transparency about limitations, and appropriate evaluation before clinical deployment. In the mid- to long-term real-world evaluations showing improved outcomes or decision quality compared with current practice will provide the most convincing basis for clinician and patient trust [64].

Alongside performance, the way AI systems present and justify their outputs also shapes trust. Explainability is frequently proposed as a solution to the ‘black box’ nature of many AI systems [65]. Post-hoc methods such as Shapley Additive Values (SHAP) can help reveal which features contributed most to a prediction and have shown promise in transplantation outcome models [9]. However, explainability is not without its challenges. Emerging evidence suggests that, when AI systems are wrong, accompanying explanations can sometimes increase clinician overconfidence and worsen decisions, an “explainability paradox” that needs to be acknowledged and considered [66]. In this context, explainability should be seen as a tool that requires evaluation and thoughtful design, rather than a substitute for robust validation and clinically meaningful evidence of benefit.

## Participatory Design

Recommendation 5: Active involvement of transplant professionals, recipients, and the public is essential at every stage of AI development, from initial concept to routine clinical implementation and evaluation.

### Participatory Design With Clinicians, Patients, and Scientists Is Essential to Ensure AI in Transplantation Addresses Real Clinical Needs

For AI systems to provide value in transplantation, they must be built around clearly defined clinical needs rather than driven by technical novelty alone. Participatory design involves clinicians, patients, scientists, and industry partners throughout the design, implementation, and evaluation of AI tools [67, 68]. Such collaboration helps identify priority use cases, specify how tools should fit into existing workflows, and anticipate unintended consequences. By avoiding siloed development and treating end users as active contributors rather than passive recipients, participatory design increases the likelihood that AI systems will be clinically relevant, usable in practice, and aligned with the priorities of the transplant community.

### Decisions About How AI Is Used in Transplantation Should Align With Patient and Public Preferences

Patient and public involvement is equally important in deciding where AI should and should not be used. Studies of transplant recipients in the UK suggest that patients are generally open to clinical use of AI and recognise its potential benefits, but are wary of unsupervised use for high-stakes tasks, favouring “human-in-the-loop” approaches in which clinicians remain clearly responsible for decisions [69]. Similar work from the wider health system indicates that patients accept AI for administrative functions such as correspondence and scheduling, but prefer its use in a decision-support role rather than as an autonomous decision-maker for diagnosis and treatment [70]. These reports underline the need to engage patient organisations in shaping AI adoption. This is also relevant to how clinical utility is defined, as the outcomes considered most meaningful may differ between stakeholders and may extend beyond traditional clinical endpoints to include equity, transparency, burden of care, and other patient-centred priorities. Aligning implementation with expressed patient and public preferences is likely to strengthen trust, improve transparency about the role of AI in care, and reduce the risk of deploying systems in ways that conflict with the values of those most affected.

## Scope and Future Directions

This position paper was intentionally developed as a broad, practical framework for the responsible adoption of AI across the transplant pathway. In prioritising breadth, multidisciplinary input, and accessibility to the wider transplant community, we did not aim to provide an exhaustive review of individual AI applications. As a result, some application areas and clinical examples could not be explored in depth, and the recommendations were developed through an iterative expert consensus process rather than a formal methodology such as Delphi. We recognise these as limitations, but also as pragmatic trade-offs that enabled the development of a clear and implementable set of recommendations spanning the major technical, ethical, regulatory, and clinical issues relevant to AI adoption in transplantation.

This paper is a starting point rather than a final or exhaustive consensus instrument. Parallel ESOT initiatives are already underway to address more focused questions relating to AI in transplantation, and these will provide opportunities to explore individual topics with greater depth and specificity.

## Conclusion/ Call to Action

The true potential of AI in transplantation will be realised through coordinated action grounded in sound scientific and ethical foundations that reflect the needs and values of our community. The scope of this work has been broad, with input from experts across multiple disciplines, and it should be viewed as a starting reference point for those wishing to explore these domains in greater depth. We urge all stakeholders to engage with these recommendations and work together to deliver on the promise of AI for transplantation.

Given the exponential development of AI technologies, these recommendations provide a basis for future refinement, keeping pace with the technology advancements while maintaining the focus on the core principles.

## Data Availability

The original contributions presented in the study are included in the article/Supplementary Material, further inquiries can be directed to the corresponding author.
