# Supplementary material for: Shaping the Future of AI in Organ Transplantation: Position Paper of the European Society for Organ Transplantation
Source: Transpl Int. 2026 Apr 20;39:16316. doi: 10.3389/ti.2026.16316 (PMC13136039; doi:10.3389/ti.2026.16316)
Supplement: Supplementary file 1 [file Supplementaryfile1.docx]

Supplementary Material – Shaping the Future of AI in Organ Transplantation: Position Paper of the European Society for Organ Transplantation (ESOT)

G Kourounis, S Gilbert, SR Knight, A Leal, JL Scully, A Loupy, DE Martin, E Preka, N Primc, FS Martinez, H Webb, G Oniscu, C Wilson

**Appendix 1**. ESOT members survey questionnaire

**Appendix 2**. ESOT members survey results

# Appendix 1. ESOT members survey questionnaire

**Question 1. How would you describe your experience with AI in transplantation or healthcare in general? *(Select single best answer)***

☐ Minimal – I have limited knowledge of AI, and do not use these tools
☐ Intermediate – I use AI-based tools but have limited understanding of how they work
☐ Advanced – I routinely use AI-based tools and have a good grasp of AI concepts
☐ Expert – I actively create or lead AI solutions in transplantation or healthcare

**Question 2. What types of AI tools do you currently use? *(Select all that apply – Required)***

☐ Large language models (e.g. ChatGPT, Claude, Gemini, DeepSeek)

☐ Generative image models (e.g. DALL·E, Stable Diffusion)

☐ Computer vision algorithms (e.g. object identification or classification)

☐ AI-powered add-ons or assistants within existing applications (e.g. Microsoft Copilot, Google AI)

☐ Other (please specify): ____________

**Question 3. When do you expect AI to have a significant impact on your clinical practice? *(Select single best answer)***

☐ It is already having a significant impact

☐ Within 5 years

☐ Within 10 years

☐ More than 10 years

☐ Never

☐ Unsure

**Question 4. Select three ESOT activities related to AI in transplantation that you believe should be top priorities. *(Select up to three options)***

☐ Develop clinical guidance and best-practice recommendations for use of AI

☐ Create clear, clinician-friendly summaries of AI regulations

☐ Define short and long-term priorities for development of AI tools for use in transplantation

☐ Establish data sharing standards to promote collaboration

☐ Promote ethical guidelines addressing biases or transparency in AI algorithms

☐ Engage with patient advocacy groups to gather patient-centred perspectives on AI adoption

☐ Set up mentoring programmes to help develop the next generation of transplant professionals working with AI

☐ Implement initiatives to improve AI literacy and trust

☐ Other (please specify): ____________

**Question 5. Select the three most significant barriers to implementing AI solutions in transplantation. *(Select up to three options)***

☐ Lack of high-quality, standardised data

☐ Lack of public trust in AI-generated clinical decisions

☐ Cost constraints or limited financial resources

☐ Regulatory and legal challenges

☐ Insufficient training or lack of AI literacy among clinicians

☐ Ethical and/or data privacy concerns

☐ Resistance to change among clinicians or administrators

☐ Bias in training data affecting equity of organ allocation and outcomes

☐ Other (please specify): ____________

**Question 6. Select three areas of transplantation where you believe AI will have the greatest impact over the next 3–5 years. *(Select up to three options)***

☐ Donor–recipient matching and organ allocation

☐ Improved predictive analytics for graft survival, organ function, or rejection risk

☐ Personalised immunosuppression regimens

☐ AI-enabled wearables for patient monitoring

☐ Organ assessment and preservation

☐ Enhanced patient engagement with specialised LLMs (e.g., trained post-transplant chatbots)

☐ AI-enabled artificial organs

☐ Other (please specify): ____________

**Question 7. Select the three potential harms associated with the use of AI in transplantation that you believe are most concerning. *(Select up to three options)***

☐ Inadvertent direct patient harm

☐ Confidential data breach

☐ Loss of clinician autonomy

☐ Risk of error/bias in decision making

☐ Loss of patient trust

☐ Loss of human jobs

☐ Other (please specify): ____________

**Question 8. Select the top three features of an AI tool that would increase your trust and willingness to use it in clinical practice. *(Select up to three options)***

☐ Transparent decision making (explainable AI)

☐ Clinical validation with peer-reviewed studies

☐ Regulatory approval (e.g. FDA, EMA)

☐ Endorsement by professional societies

☐ Accuracy and predictive performance metrics

☐ High data security and patient confidentiality

☐ Ease of access (available in App stores)

☐ Integration with electronic health records

☐ Recommendation/endorsement by colleagues/employer

☐ Other (please specify): ____________

**Question 9. Select the top three benefits you believe AI could bring to your practice as a transplant professional. *(Select up to three options)***

☐ Increased diagnostic accuracy

☐ Increased transplant success rate

☐ Reduced medical errors

☐ Improved patient experience of care

☐ Increased patient autonomy

☐ More efficient use of resources including clinician time

☐ Improved equity of access to transplantation

☐ Other (please specify): ____________

# Appendix 2. ESOT members survey results

The questionnaire was open between 14/07/2025 and 14/10/2025, and it was completed by 57 members. The questions and responses were grouped into three categories: Members’ experience of AI and highest priorities for future development, anticipated areas of most benefit and impact for organ transplantation, and areas of risk and concern surrounding the development of AI.

## Members’ experience and priorities

Most respondents identified their AI experience as intermediate (40.4%), meaning that they use AI tools but have a limited understanding in how they work. The most reported AI tools used by members were large language models (LLMs), used by 89.5% of respondents, and Copilot applications, used by 38.6% of respondents. Additional tools, reported in the ‘Other’ category, included the iBox and ambient scribing services for consultations. The three ESOT AI-related activities in transplantation that members identified as top priorities were the development of clinical guidance and best practice recommendations for the use of AI, the defining of short and long term priorities in the future development of AI applications in transplantation, and the promotion of ethical guidelines addressing biases and transparency in AI algorithms. A detailed breakdown of these results is presented in Figure S1.

Figure S1. Survey responses on AI experience, tool use, and ESOT AI-related priorities in transplantation.

## Benefits and impact

On the anticipated benefits and timelines of adoption, most respondents expect AI to have a significant impact on their clinical practice within <5 years (89.5%). The transplantation domains anticipated to experience the greatest impact were donor–recipient matching and organ allocation (82.5%), improved predictive analytics (71.9%), and the use of AI-enabled wearables for patient monitoring (43.9%). The top three benefits respondents expected from AI were greater diagnostic accuracy (64.9%), enhanced resource efficiency (61.4%), and fewer medical errors (49.1%). A breakdown of the full results is presented in Figure S2.

Figure S2. Survey findings on anticipated impact and benefits of AI in transplantation.

## Risks and concerns

On the topic of anticipated risks and barriers, the three most frequently selected were regulatory and legal challenges (54.4%), paucity of high-quality standardised data (50.9%), and lack of AI literacy among clinicians (47.4%). The top three reported potential unintended harms associated with AI in transplantation were the risk of error or bias in decision making (80.7%), inadvertent direct patient harm (59.6%), and confidential data breaches (52.6%). Finally, the features most likely to increase trust and willingness to adopt AI in clinical practice were transparent computational decision-making with explainable AI (XAI) (61.4%), clinical validation through peer-reviewed studies (57.9%), and successful regulatory approval (40.4%). A breakdown of the full results is presented in Figure S3.

Figure S3. Survey findings on barriers and concerns for AI adoption, and features to improve trust.
